# Statin the course: Navigating unchartered territory in cirrhosis

**DOI:** 10.1097/HC9.0000000000000456

**Published:** 2024-06-03

**Authors:** Camille A. Kezer, Kathryn A. Schmidt, Vijay H. Shah

**Affiliations:** Department of Internal Medicine, Division of Gastroenterology and Hepatology Mayo Clinic 200 First Street, SW Rochester, Minnesota, USA

## WHY STATINS?

**FIGURE 1:**
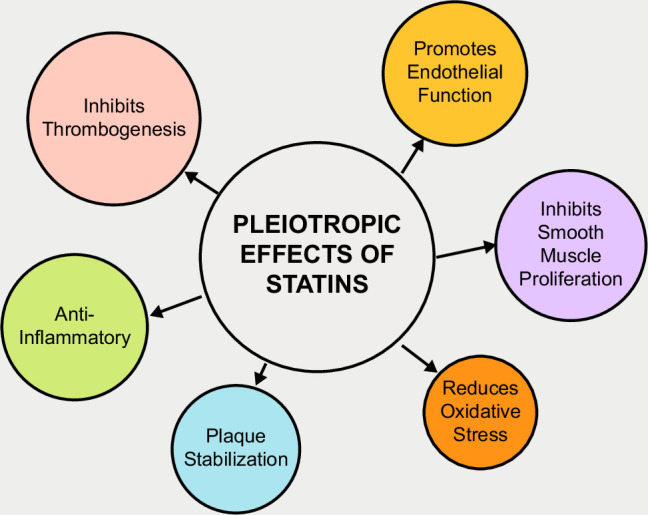
Pleiotropic effects of statins.

Cirrhosis is a disease of significant morbidity and mortality worldwide, and current treatment is focused on managing sequela.^1^ There are no existing medications to prevent or reverse hepatic decompensation, but statins are under investigation as a potential therapeutic agent for cirrhosis.^1^ Statins act by inhibiting 3-hydroxy-3-methylglutaryl CoA reductase.^2^ The potential benefits of statins in cirrhosis stem from their pleiotropic effects, Figure 1. including modulation of endothelial cell function, suppression of HSC activation, and the inhibition of thrombogenesis in blood cells.^3^ Collectively, these mechanisms target the deleterious pathophysiology of portal hypertension by promoting vasodilation, reducing intrahepatic vascular resistance, and inhibiting fibrogenesis. Through these mechanisms, statins have the potential to reduce the risk of hepatic decompensation and complications associated with portal hypertension.^4^ Additionally, statins have anti-inflammatory effects that could reduce liver disease progression. Statin effects are mediated through inhibition of RhoA and Ras signaling cascades.^4^ For these reasons, statins are an attractive therapeutic candidate for patients with cirrhosis.

## WHAT STATINS DO

Both preclinical and clinical studies have shown a variety of potential benefits of statin therapy in the treatment of chronic liver disease. A large systematic review and meta-analysis found that statin use was associated with a 46% lower risk of hepatic decompensation and a 46% reduction in mortality in patients with chronic liver disease.^1^ However, for their primary indication of cardiovascular risk reduction, statins are often under-prescribed in patients with chronic liver disease due to clinicians’ concerns about hepatotoxicity.^2^ Statins have been implicated as a cause of DILI, but this is extremely rare.^2^,^5^ Simvastatin at higher doses has been associated with increased liver and muscle toxicity in patients with advanced decompensated cirrhosis.^6^ Much of the current literature supporting the benefits of statins is observational in nature with heterogeneity in certain statin-related outcomes,^1^ and carries a high risk of residual confounding.^7^ Randomized trials are needed to further elucidate the impact of statins on patients with cirrhosis.

## THE STATLIVER TRIAL

In efforts to clarify the potential of statins, Kronborg et al^8^ conducted the StatLiver trial, a multicenter, randomized, placebo-controlled trial of adults with cirrhosis with clinically significant portal hypertension. Their mission? To investigate the safety of atorvastatin and its effects on survival, hospital admissions, systemic inflammation, and lipidomics compared with placebo. Of the 78 participants recruited, 59 participants completed > 180 days of the intervention. Atorvastatin failed to significantly alter the mortality or reduce hospital admissions in patients with cirrhosis with clinically significant portal hypertension. There were also no differences between the groups in portal hypertension as assessed by transjugular vein catheterization, Model for End-Stage Liver Disease, and biochemical parameters over 6 months. The authors found differences in 3 of 42 inflammatory markers, CD62-L-selectin (CD62L), matrix metalloproteinase 2, and TNF-α, and identified changes in 20 lipid classes, primarily in triglycerides and hexosylceramides. However, when adjusting for multiple testing, there were no significant differences in lipidomics between the groups. Atorvastatin appeared safe, with no patients in the atorvastatin group experiencing toxic hepatitis, serious treatment-related adverse events, or death.

## EXPLAINING UNEXPECTED RESULTS

We suspect the difference between the StatLiver trial results and prior observational data is explained by the nuanced interplay between statins and the dynamic progression of liver disease and limitations due to potential confounding variables, sample size, and follow-up duration. It is also worth considering the role immortal time bias may play in this study given the observational data; this bias may occur in observational studies when the follow-up time for 1 group of subjects is incorrectly classified as unexposed time when, in fact, they could not have experienced the outcome of interest due to the study design, creating the illusion of a protective effect for the exposure being studied.

Future research is needed to enhance our understanding of statins in cirrhosis with a focus on cirrhosis-related outcomes, including decompensation and mortality. Ideally, larger randomized trials inclusive of diverse patients with different etiologies of chronic liver disease, various stages of the disease, and across multiple centers are needed. Consideration of statin dosage, statin type, and long-term follow-up will also be crucial in elucidating whether statins can truly affect patient outcomes and decrease liver-related mortality.

## THE STATLIVER TRIAL IN CONTEXT

There are at least 3 other trials of statins. First, the LiverHope trial explored the impact of statins in a randomized, double-blind, placebo-controlled study, where patients received either simvastatin 40 mg/day or 20 mg/day plus rifaximin 1200 mg/day in both arms. In a preliminary study, the investigators explored the effects on plasma metabolites associated with acute-on-chronic liver failure. Treatment with simvastatin and rifaximin led to alterations in 161 of 985 metabolites compared to placebo treatment, notably reducing levels of metabolites associated with the tryptophan–kynurenine and carnitine pathways, which are associated with inflammation and mitochondrial dysfunction. Though this study sheds light on the mechanisms underlying the effects of these treatments in decompensated cirrhosis and suggests the potential use of metabolomics in investigating targeted therapies to prevent acute-on-chronic liver failure in cirrhosis,^9^ the larger LiverHope trial did not find any benefits from simvastatin and rifaximin therapy.

Second, there is an ongoing multicenter double-blind phase II study assessing the safety and efficacy of rosuvastatin compared to placebo in mean-change in liver stiffness from baseline to end of the study period at 2 years in patients with compensated cirrhosis.^10^ Third, there is an ongoing phase III clinical trial designed to investigate the potential of simvastatin 40 mg/day to reduce the risk of hepatic decompensation among US Veterans with compensated cirrhosis over a 2-year period. The study will additionally assess the impact of genetic variations on the safety and efficacy of statin therapy and examine its influence on patients’ quality of life.^11^

## CONCLUSION

The StatLiver trial demonstrates that while statins are safe and overall, well tolerated in cirrhosis, they do not appear to have clinical benefit, at least not at the 6-month time interval. As the enrolled patients could be compensated or decompensated, the heterogeneity of the patient population may limit our understanding of the optimal timing of initiating statins. Amidst the maze of cirrhosis management, the StatLiver trial serves not as a conclusion but as a prologue to further inquiry. The StatLiver trial has shed light on the potential role of statins in clinical and exploratory end points. Further studies looking at additional clinical end points over time, such as changes in biomarkers, liver stiffness, portal hypertension, and transplant-free survival, are needed to determine the potential benefits of statin therapy.
